# Transcriptomic Characterization of Nitrate-Enhanced Stevioside Glycoside Synthesis in Stevia (*Stevia rebaudiana*) Bertoni

**DOI:** 10.3390/ijms22168549

**Published:** 2021-08-09

**Authors:** Yuming Sun, Ting Zhang, Xiaoyang Xu, Yongheng Yang, Haiying Tong, Luis Alejandro Jose Mur, Haiyan Yuan

**Affiliations:** 1 Jiangsu Key Laboratory for the Research and Utilization of Plant Resources, Institute of Botany, Jiangsu Province and Chinese Academy of Sciences, No. 1 Qianhuhoucun Village, Zhongshan Gate, Nanjing 210014, China; yumingsun@cnbg.net (Y.S.); zhangting901014@cnbg.net (T.Z.); xuxiaoyang@cnbg.net (X.X.); yongheng@cnbg.net (Y.Y.); haiyingtong@cnbg.net (H.T.); 2 The Jiangsu Provincial Platform for Conservation and Utilization of Agricultural Germplasm, Nanjing 210014, China; 3 Institute of Biological, Environmental and Rural Sciences, Aberystwyth University, Aberystwyth SY23 3DA, UK; lum@aber.ac.uk

**Keywords:** *Stevia rebaudiana*, nitrogen forms, transcriptome, secondary metabolism, transcription factors

## Abstract

Nitrogen forms (nitrate (NO_3_^−^) or ammonium (NH_4_^+^)) are vital to plant growth and metabolism. In stevia (*Stevia rebaudiana*), it is important to assess whether nitrogen forms can influence the synthesis of the high-value terpene metabolites-steviol glycosides (SGs), together with the underlying mechanisms. Field and pot experiments were performed where stevia plants were fertilized with either NO_3_^−^ or NH_4_^+^ nutrition to the same level of nitrogen. Physiological measurements suggested that nitrogen forms had no significant impact on biomass and the total nitrogen content of stevia leaves, but NO_3_^−^-enhanced leaf SGs contents. Transcriptomic analysis identified 397 genes that were differentially expressed (DEGs) between NO_3_^−^ and NH_4_^+^ treatments. Assessment of the DEGs highlighted the responses in secondary metabolism, particularly in terpenoid metabolism, to nitrogen forms. Further examinations of the expression patterns of SGs synthesis-related genes and potential transcription factors suggested that GGPPS and CPS genes, as well as the WRKY and MYB transcription factors, could be driving N form-regulated SG synthesis. We concluded that NO_3_^−^, rather than NH_4_^+^, can promote leaf SG synthesis via the NO_3_^−^-MYB/WRKY-GGPPS/CPS module. Our study suggests that insights into the molecular mechanism of how SG synthesis can be affected by nitrogen forms.

## 1. Introduction

As the global standard of living improves, people are aiming to pursue a healthier lifestyle. The excessive intake of sugars has increased the risk of dental caries, diabetes, obesity and hyperlipidemia, so that the alternative new sugar crop, stevia (*Stevia rebaudiana* Bertoni), has received increasing attention and acceptance [1,2]. Stevia plants are rich in steviol glycosides (SGs), a class of tetracyclic diterpenoid compounds, especially in leaf tissue [3]. Stevioside (STV) and rebaudiosides A (Reb A) are the most abundant SGs and can account for about 4–20% of the dry leaf weight. Other less abundant SGs, such as Reb C, Reb F and Dulcoside A, may be important in providing taste to SG mixtures [4]. SGs can be used as food additives and are sweeter than cane sugar and beet sugar, but have a lesser calorific value. These properties have greatly increased worldwide demand for SGs and resulted in increased commercial cultivation of stevia plants [5,6]. Given this, improving the SG levels in stevia plants through agricultural practices is of vital importance for the development of the stevia industry.

SG metabolism in stevia plants may be influenced by multiple agricultural factors such as planting density, water management, arbuscular mycorrhiza infections and nutrition [7,8,9]. Amongst these, nitrogen (N) nutrition is one of the most important factors that influence stevia productivity, as N is deeply implicated in plant development as well as metabolic processes [10,11]. Previous studies, including ours, have shown that N fertilization can enhance leaf photosynthesis rate and aboveground biomass, but, if improperly used, may inhibit SG synthesis and accumulation [12,13,14,15]. Thus, we found that SG levels can be increased to a certain extent through optimizing N topdressing strategies [16]. However, there was a negative correlation between SG synthesis and leaf N content, as well as the leaf biomass under different N fertilization rates and topdressing strategies. Recently published transcriptome work attributed the N-inhibited SG metabolism to either shift in the balance of commitment to plant growth versus differentiation or transcriptionally repressive events after adding N [17]. This work highlights the need for a subtle implementation of N fertilization rates or timing to promote biomass but limit negative effects on the levels of SGs.

The major forms of mineral N, ammonium (NH_4_^+^) and nitrate (NO_3_^−^) are differently absorbed, assimilated, and they also have differential impacts on plant metabolism. The assimilatory costs of NH_4_^+^ are lower than NO_3_^−^ because it can be directly assimilated in root tissue, whilst NO_3_^−^ needs to be transported to the leaves for further reduction to NH_4_^+^ [18,19]. The differential effects of NO_3_^−^ have been linked to leaf photorespiration and the tricarboxylic acid cycle to affect other central metabolic pathways [20,21]. Further, an association between NH_4_^+^ assimilation and phosphoenolpyruvate carboxylase activity involves altered carbon (C) and energetics metabolism [22,23]. Crucially, the form of N affects the terpenoid metabolism of plant. A higher NO_3_^−^/NH_4_^+^ ratio has been shown to significantly promote terpenoid synthesis in terpenoid-rich plants such as *Brassica* species [24] and *Prunella vulgaris* [25]. However, opposite results have also been documented in *Andrographis paniculata* [26], *Capsicum annuum* [27] and *Occimum basilicum* [28]. Thus, N forms can reshape plant terpenoid metabolism, but the exact action may differ between plant species. It is therefore important to investigate the relationship between N forms and SGs metabolism in stevia leaves, as well as define the underlying mechanisms.

In the current study, we combined pot and field experiments to demonstrate that NO_3_^−^ rather than NH_4_^+^ fertilization can significantly increase leaf SG contents without influencing leaf biomass formation. Via further transcriptomic analysis, we found that such effects are likely due to the upregulation of the terpenoid synthesis pathway by NO_3_^−^ treatment. This NO_3_^−^-induced change could be attributed to the transcription factors belonging to MYB and/or WRKY families. Our results have implications for how SGs synthesis can be maximized in the field-grown stevia.

## 2. Results

### 2.1. Effects of Nitrogen Forms on the Biomass, Carbon–Nitrogen Status and SGs Content in the Leaves of Stevia Plants Grown under Pot and Field Conditions

Different forms of N fertilization ((NH_4_^+^) vs. (NO_3_^−^)) did not significantly affect the biomass of stevia leaves. This was reflected in the similarities in TN, TC and C/N ratio between NH_4_^+^- and NO_3_^−^-fed stevia plants (Table 1). However, whilst leaf NH_4_^+^ content was not significantly changed by N forms, NO_3_^−^ content was significantly increased in NO_3_^−^ treatments, under both pot and field conditions.

**Figure 1:**
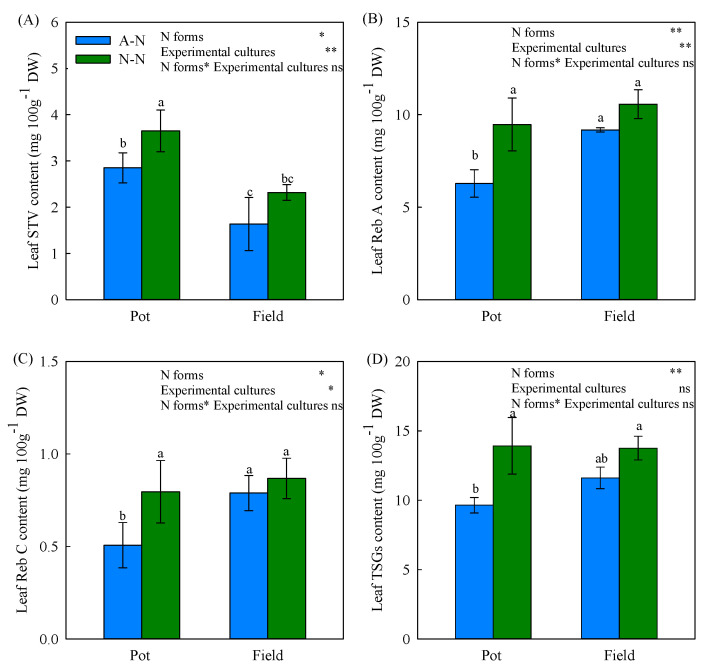
Effect of nitrogen forms on the leaf contents of stevioside (STV, **A**), rebaudioside A (Reb A, **B**), rebaudioside C (Reb C, **C**) and total steviol glycosides (TSGs, **D**) of stevia plant growing in pot and field conditions (“experimental cultures”). Stevia plants growth under pot or field conditions were fed with ammonium–nitrogen (A-N) or nitrate–nitrogen (N-N) to the same N level. Values represent the means ± SD of three biological replicates. ANOVA results are indicated; different letters indicate significant differences in the same indicator, *, ** and ns indicate significant difference at 0.05, 0.01 probability levels and nonsignificant difference, respectively.

N application forms significantly altered the contents of leaf SGs. The major SG in stevia plants, Reb-A, was considerably increased by NO_3_^−^ by 50.79% and 15.14% under pot and field conditions, respectively, compared to NH_4_^+^ treatment (Figure 1A). The leaf contents of STV and Reb-C were also increased by NO_3_^−^ rather than NH_4_^+^ treatment (Figure 1B,C). However, total SGs (TSGs) contents were significantly greater in NO_3_^−^-fed compared to NH_4_^+^-fed plants (Figure 1D). When examining the effects of the “experimental cultures” used, a higher leaf STV content but lower Reb-A and Reb-C contents were observed in stevia plants grown in pot than field culture. No significant interaction effect was exhibited in the content of either a single SG or TSGs.

### 2.2. Global Analysis of RNA-Seq Data

Stevia leaves treated by different N forms were sampled and sequenced for transcriptome analysis. Approximately 21,444,479 and 25,727,058 clean reads were obtained from NH_4_^+^-and NO_3_^−^-treated samples, which corresponded to 6.41 GB and 7.70 GB of data (Supplemental Table S1), respectively. In addition, the frequency of >30 Phred quality score (Q30) was higher than 94.27% and the guanine–cytosine (GC) content was higher than 45.42% for all the samples, indicating that the sequence data were of high quality. Then, 60.45–76.30% of the clean reads were mapped to the reference genome of stevia plants (https://doi.org/10.6084/m9.figshare.14169491.v1 (accessed on 5 March 2021)) [29], and 48.83–62.55% of the clean reads were uniquely mapped onto the stevia genome. In total, 35,424 and 36,063 genes were expressed (FPKM > 0) in the A-N and N-N treatments, respectively (Supplemental Table S1).

### 2.3. Identification of DEGs Responsive to N Forms

**Figure 2:**
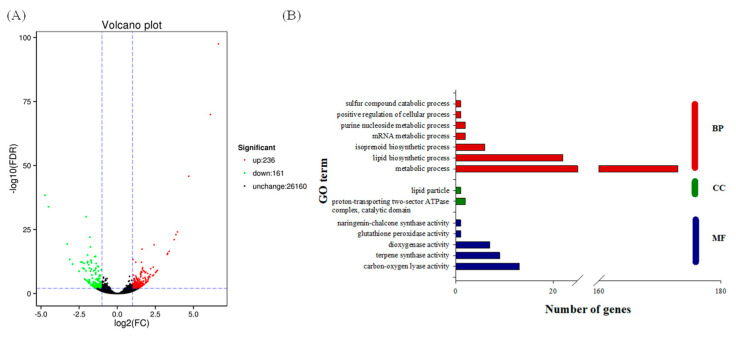
Volcano plot (**A**) and gene ontology (GO, **B**) analysis of differentially expressed genes (DEGs) between ammonium (A-N)- and nitrate (N-N)- treated stevia leaves. In (**A**), the red dots represent the upregulated DEGs, while the green dots represent the downregulated DEGs, with a minimum fold-change threshold of 2 and a significant level of 0.05. In (**B**), the *y*-axis represents different GO terms that belong to BP (biological processes), CC (cell component) or MF (molecular functions) subcategories, while the value on the *x*-axis shows the number of genes in the corresponding GO term.

To identify the DEGs’ specific response to N forms, the calculations were based on FPKM outputs where a fold change ≥ 2 and false discovery rate (FDR) < 0.01 were used as thresholds to be passed. A total of 397 DEGs were identified, with 236 upregulated genes and 161 downregulated genes (Figure 2A). To explore the functions of these genes, the DEG sets were assigned to 36 functional groups by gene ontology (GO) annotation analysis, including “biological process” (BP, 14 subcategories), “cellular component” (CC, 11 subcategories) and “molecular function” (MF, 11 subcategories) (Supplemental Figure S1). In the BP group, the majority of GO terms were linked to metabolic process, cellular process and single-organism process. For the CC group, the top five subcategories were membrane, cell, cell part, membrane part and organelle. Catalytic activity and binding were the dominant subcategories in the MF group.

GO enrichment analysis was performed to characterize the main biological functions of DEGs. This indicated that metabolic process (GO: 0008152), lipid biosynthesis process (GO: 0008610) and isoprenoid biosynthetic process (GO: 0008299) were the most significant enrichment terms in the BP category, whilst the carbon–oxygen lyase activity (GO: 0016835) and terpene synthase activity (GO: 0010333) were enriched in the MF category (Figure 2B).

**Figure 3:**
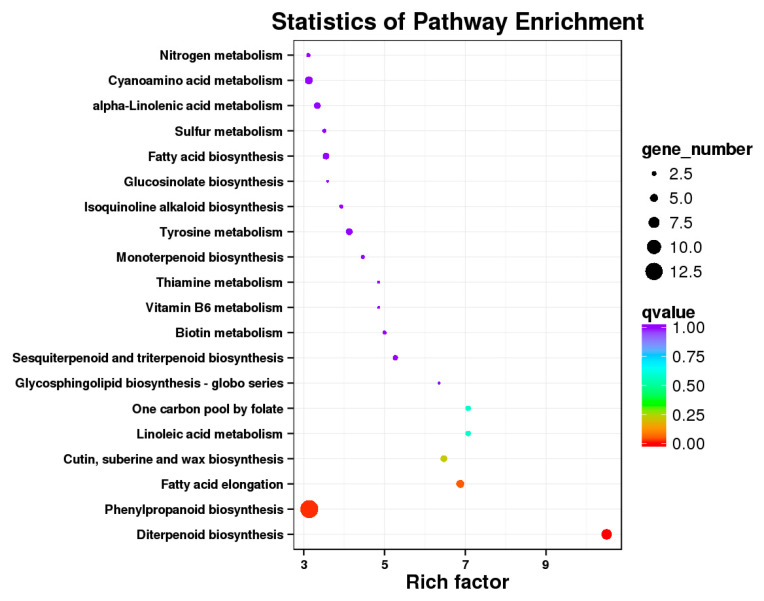
KEGG (Kyoto encyclopedia of genes and genomes, C) pathway enrichment analysis of differentially expressed genes (DEGs) between the two groups (ammonium (A-N) vs. nitrate (N-N)). The *x*-axis indicates the enrichment factor, while the color of each circle relates to the enriched Q-value and the size is equivalent to the gene numbers mapped to the pathway.

KEGG enrichment analysis was then conducted to assign the DEGs to cellular pathways. Interestingly, these DEGs were enriched in pathways including “diterpenoid biosynthesis”, “phenylpropanoid biosynthesis”, “fatty acid elongation” and “cutin, suberine and wax biosynthesis”, following NO_3_^−^ rather than NH_4_^+^ treatment (Figure 3, Supplemental Figure S2A). When considering those DEGs downregulated by NO_3_^−^, the “carbon metabolism” together with “amino acid metabolism” was prominent (Supplemental Figure S2B).

### 2.4. MapMan Analysis

**Figure 4:**
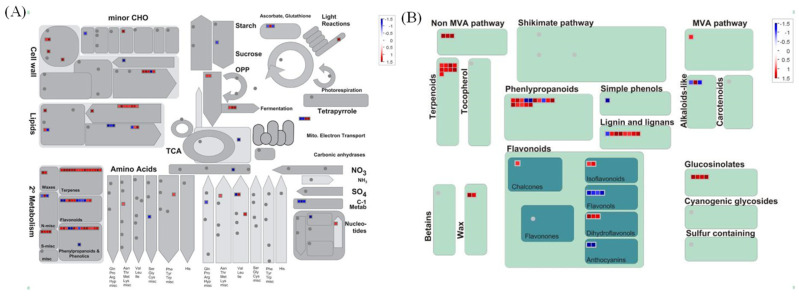
Mapping genes on overview map (**A**) and a secondary metabolism map (**B**) that were differentially expressed in nitrate (N-N)-treated in comparison to ammonium (A-N)-treated stevia leaves.

To investigate the metabolic pathways implicated in the response to N forms from a global perspective, we analyzed 397 DEGs using MapMan analysis. Most of the DEGs were assigned to four different metabolic pathways, including “cell wall”, “lipids”, “secondary metabolism” and “amino acids” (Figure 4A). Then, we specifically analyzed the response of secondary metabolism, which is closely related to SGs’ synthesis. Interestingly, genes involved in terpenoid synthesis and phenylpropanoids and lignin and lignans’ metabolism were significantly enhanced by NO_3_^−^ nutrition when compared with NH_4_^+^ nutrition (Figure 4B). SGs biosynthesis mainly contains four modules after the glycolysis processes, which are methylerythritol 4-phosphate (MEP) module, terpene synthesis module, cytochrome P450 module and glycosylation module (Supplemental Figure S3) [5]. In our MapMan assessment, we observed significantly enhanced expressions of genes involved in the MEP pathway.

### 2.5. Effect of Nitrogen Forms on the Expression of Genes-Encoding SG Synthesis in Stevia Leaves

**Figure 5:**
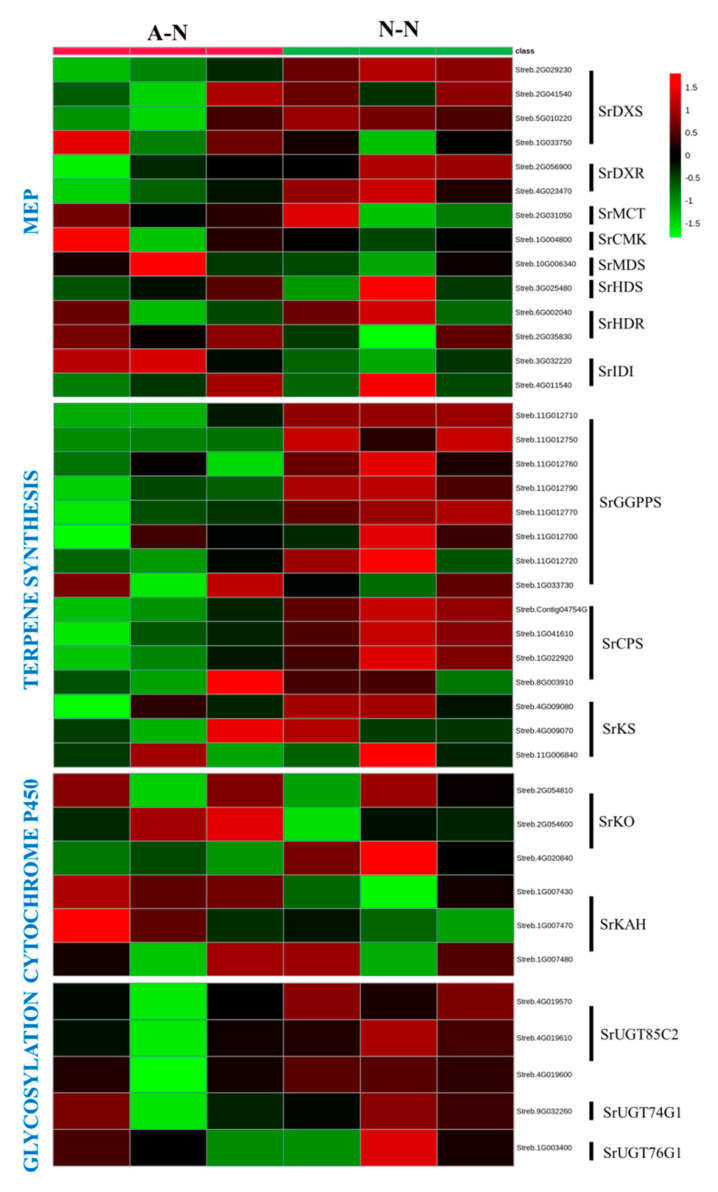
Heatmap showing the expression profiles of the genes involved in steviol glycosides synthesis in ammonium (A-N)- and nitrate (N-N)-treated stevia leaves.

We next focused on the expression of specific genes in the MEP module (Supplemental Figure S3). The expression of genes encoding 1-deoxy-D-xylulose-5-phosphate synthase (DXS) and 1-deoxy-D-xylulose 5-phosphate reductoisomerase (DXR) were higher under NO_3_^−^ nutrition than NH_4_^+^ nutrition (Figure 5). However, the expressions of other genes involved in the MEP module did not significantly differ between NH_4_^+^- and NO_3_^−^-fed plants. Genes encoding geranylgeranyl pyrophosphate synthase (GGPPS) and ent-copalylpyrophosphate synthase (CPS) in the terpene synthesis module, as well as the genes encoding UDP-glycosyltransferase 85C2 (UGT85C2) in the glycosylation module, were upregulated by NO_3_^−^ treatments compared to NH_4_^+^. In contrast, those encoding ent-copalyl diphosphate synthase (KS), UDP-glycosyltransferase 74G1 (UGT74G1) and UDP-glycosyltransferase 76G1 (UGT76G1) were not changed by N forms (Figure 5). No significant difference was observed for the genes encoding cytochrome P450 enzymes, ent-kaurene oxidase (KO) and kaurenoic acid hydroxylase (KAH) when comparing the two treatments.

### 2.6. Analysis of Transcription Factors (TFs) Responses to N Forms

**Figure 6:**
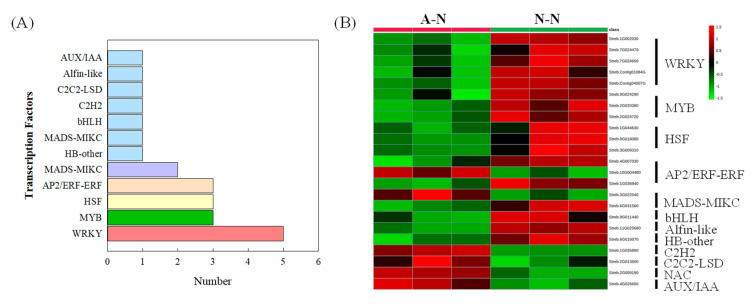
Transcription factors identified from the DEGs between ammonium (A-N)- and nitrate (N-N)-treated stevia (**A**) and a heatmap showing their expression profiles (**B**).

We next analyzed the expression of TFs to fully understand the mechanism of NO_3_^−^-affected terpenoid biosynthesis. A total of 23 differentially expressed TFs were identified by comparative analysis between NH_4_^+^- and NO_3_^−^-fed plants. Among these TFs, WRKY (5), MYB (3), HSF (3) and AP2/ERF-ERF (3) were most prominent (Figure 6). NO_3_^−^ treatment positively regulated the expression of genes relating to the WRKY, MYB and HSF families, while opposite results were observed for AP2/ERF-ERF family genes.

### 2.7. Validation of Gene Expression Patterns

**Figure 7:**
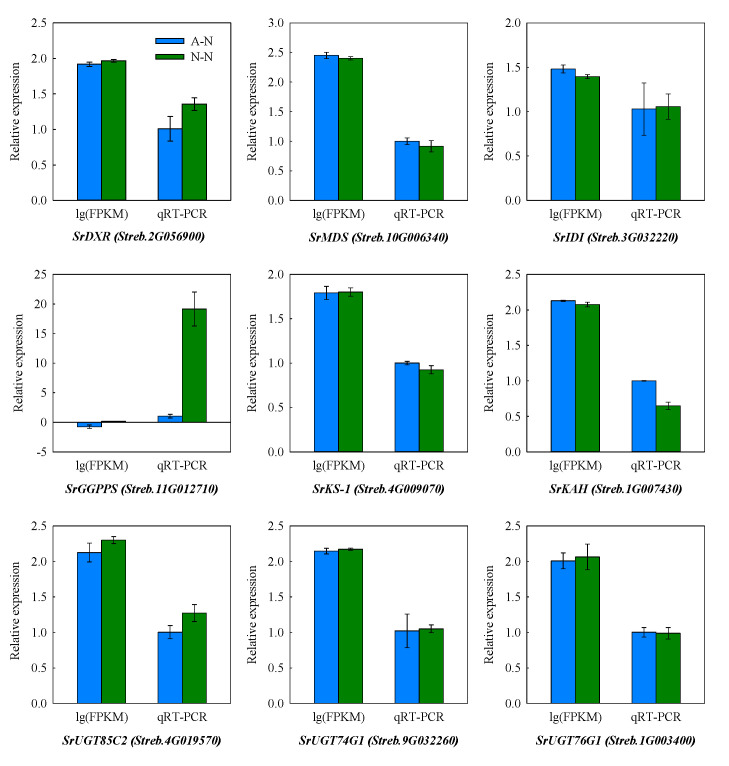
Quantitative real-time PCR (qRT-PCR) assessments of 9 genes encoding the key enzymes involving in steviol glycosides synthesis. Stevia plants were fed with either the ammonium form of nitrogen (A-N) or the nitrate form of nitrogen (N-N). Values represent the mean ± SD of three biological replicates.

To validate some key observations from the RNA-seq data, the expression of nine genes involving in the SG biosynthesis was analyzed by qRT-PCR. Both the RNA-Seq and qRT-PCR results showed that NO_3_^−^ nutrition significantly increased the expression levels of *SrDXR* (Streb.2G056900), *GGPPS* (Streb.11G012710) and *UGT85C2* (Streb.4G019570), while the expressions of *SrMDS* (Streb.10G006340) and *SrKAH* (Streb.1G007430) were reduced by NO_3_^−^ treatment compared to NH_4_^+^ (Figure 7). No significant difference was observed in other genes in either of the two methods. Taken together, the comparative analysis of the expression patterns of these genes from RNA-Seq and qRT-PCR results showed a good match, which suggested the reliability of the RNA-Seq results used in this study.

## 3. Discussion

The absorption of N by plants is critical for plant growth but incorporates preferences for different forms of N to influence metabolic programming. Thus, supply of different N forms will alter plant N assimilation process, C fixation efficiency and subsequently the formation of biomass [30,31]. More specifically, the metabolism of specific plant compounds, including terpenoids, can be influenced by N forms. For instance, the production of andrographolide in *Andrographis paniculata* [26] and taxol in *Taxus yunnanensis* [32] could be enhanced by NH_4_^+^ nutrition when compared to NO_3_^−^. In contrast, NO_3_^−^ nutrition, rather than NH_4_^+^, increased the synthesis of periplocin in *Periploca sepium* [33], ginseng saponin in *Panax quinquefolium* [34] and essential oil in *Anethum graveolens* [35]. This was in line with our data, whereby NO_3_^−^ was linked to a higher leaf SGs contents that could be linked to elevated expression of SGs synthesis genes (Figure 1 and Figure 5). However, Qin, et al. [36] found that the relationship between NH_4_^+^/NO_3_^−^ ratios and triterpenoid accumulation in *Cyclocarya paliurus* was influenced by sampling time or the organs being tested [36]. This was also implied in a recent study based on stevia, where different N form-induced effects on SGs were observed when supplied with different plant growth regulators [37]. Taking all of these observations together, although N form-regulated terpenoid metabolism can be influenced by species and the organ being tested, there appears to be a positive role of NO_3_^−^ nutrition in SGs synthesis.

We have previously demonstrated that application of N as urea boosted biomass but this effectively “diluted” SGs content [15]. However, in this present work, different forms of N did not result in a significant difference in either leaf N content or biomass (Table 1), but there was higher SGs content in NO_3_^−^-treated plants (Figure 1). Crucially, this could reduce any dilution effect of growth on the levels of SGs. To further elucidate the underlying mechanism for this, transcriptomic analysis was performed. Further, we validated the RNA-seq data through qRT-PCR detection, as also used in many other studies [38,39,40,41]. Accordingly, the similar expression patterns of specific SG synthesis-related genes from RNA-Seq and qRT-PCR results confirmed the validity of our transcriptome analysis results (Figure 7). These transcriptomic analyses suggested the key genes, TFs and pathways in regulating specific metabolism, most of which have been previously reported in terpenoid-enriched plants including Finger Citron [42], *Ferula assafoetida* [43], *Dendrobium officinale* [44], as well as stevia [17,45]. The KEGG results clearly indicated that NO_3_^−^ mostly upregulates the pathways relating to secondary metabolism, with “diterpenoid biosynthesis” being the most significant for SGs. Other important pathways were “phenylpropanoid biosynthesis” and “wax biosynthesis”, both of which were also supported by the MapMan analysis (Figure 3 and Figure 4). Such results imply that N forms play a definite regulatory role in metabolic reprogramming, which has been documented in poplar [46] and tea [47]. NH_4_^+^ upregulates the primary amino acid metabolism as well as the secondary alkaloids metabolism, which would correspondingly reduce the metabolic flow to terpenoids [48,49]. This aligned with our results that amino acid synthesis-related pathways prominent in NO_3_^−^ downregulated DEGs (Supplemental Figure S2).

Interestingly, different modules of SG biosynthesis responded differently to N forms (Figure 5). For instance, the expressions of genes in the P450 and glycosylation modules were not significantly affected by N form, which suggest that these genes were not driving the increases in NO_3_^−^-mediated SGs synthesis, although they play vital roles in either terpenoid skeleton modification or glycosylation [50]. In contrast, the SrDXS and SrDXR families were upregulated by NO_3_^−^, suggesting that these genes contributed to increased SGs synthesis, with their function in mediating metabolic flow into the MEP pathway. This aligns with MEP being the main pathway in providing the 5-carbon isoprenoid unit for SG biosynthesis [29,51]. Even more striking was the upregulation of genes encoding GGPPS and CPS, by NO_3_^−^ feeding. This was an important observation as these are the key catalytic enzymes responsible for ent-diterpenoid synthesis and are highly conserved in all terpenoid-synthesizing organisms [52]. Thus, enhanced diterpene synthesis and accumulation have been achieved by overexpressing the CPS or GGPPS gene in *Salvia* species [52,53] and tobacco [54]. Similarly, Jassbi et al. [55] demonstrated that the silencing of *GGPPS* disrupted the synthesis of 17-hydroxygeranyllinalool diterpenoid glycosides, together with the increased susceptibility to tobacco hornworm. Overall, the above findings indicated that the NO_3_^−^-mediated SG biosynthesis was related to the upregulated terpenoid biosynthetic genes, especially GGPPS and CPS.

Beyond demonstrating some upregulated terpenoid biosynthetic gene expression, we sought to suggest some key NO_3_^−^ responsive regulatory components. In defining these, TFs were the obvious targets as these are key factors determining plant secondary metabolism in response to environmental changes (including N forms) and activating the promoters of specific genes [56,57]. In this study, 23 TFs were screened and WRKY and MYB TFs were the most enriched and positively responded to NO_3_^−^ (Figure 6). Notably, it seemed that all previously documented WRKY TFs play positive functions in endogenous terpenoid biosynthesis (Supplemental Table S2). This aligns with reports that WRKY TFs regulate plant defence via secondary metabolism regulation [58,59]. Moreover, studies in *Salvia miltiorrhiza*, *Panax ginseng* and *Withania somnifera* all show that WRKY TFs can positively direct terpenoid biosynthesis, by binding to the W-box sequences in promoters of specific genes, such as *CPS* [60], *squalene epoxidase* [59,61] and *DXR* [62]. Interestingly, Sun et al. [63] demonstrated that the PqWRKY1 in *Panax quinquefolius*, that associated with the increased production of ginsenosides, can also upregulate triterpene biosynthetic genes in *Arabidopsis*, indicating the conserved functions of WRKY across different plant species. In short, the above findings support a positive role of WRKY TFs in the synthesis of terpenoids, including SGs in stevia plants. Considering MYB TFs, they have been reported to play key roles in plant growth, defence, as well as secondary metabolism. Although MYB TFs may regulate terpenoid biosynthesis as either activators or repressors (Supplemental Table S3), it seems that they have a positive regulatory effect in NO_3_^−^-mediated SGs synthesis, which was also confirmed in our more recent work [17]. Terpenoid-relevant MYB targeted promoters include those encoding GGPPS [64], dammarenediol synthase [65], CPS [66] and other TPS genes [67,68]. Other TFs such as AP2/ERF-ERF and bHLH have also been reported to play regulatory roles in terpenoid synthesis [69,70], but we could find no evidence of these being significant factors mediating N form-mediated metabolic reprogramming. Further, although HSF TFs were upregulated by NO_3_^−^ rather than NH_4_^+^, rather than affecting plant secondary metabolism, they seem to be mainly regulated plant biotic/abiotic stress responses through various signalling pathways such as calcium, reactive oxygen species and abscisic acid [71,72]. Taking all of these observations into account, we suggest that WRKY and MYB TFs were the key factors responding to N forms and mediating the synthesis of terpenoid compounds, including SGs in our study.

**Figure 8:**
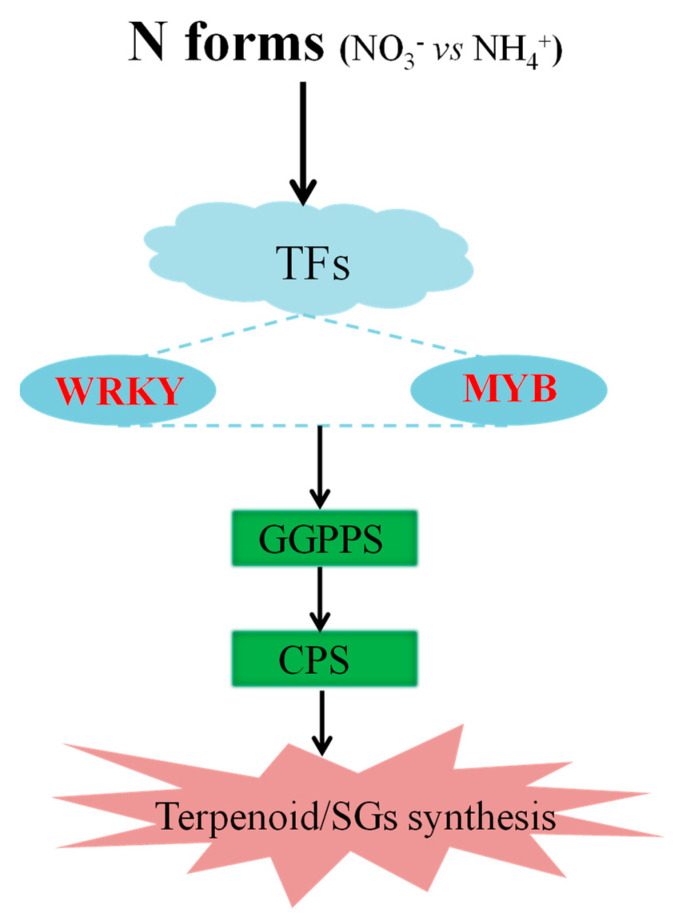
A schematic model showing the regulation of steviol glycosides synthesis by N forms-MYB/WRKY-GGPPS/CPS in stevia leaves. When feeding with nitrate nitrogen (N-N) rather than ammonium nitrogen (A-N), specific transcription factors in stevia leaves, especially those belonging to MYB and WRKY families, are activated. Theses TFs can regulate the expressions of terpene synthesis-related genes such as GGPPS and CPS, thereby promoting the production of steviol glycosides.

To summarize, we have derived a schematic model to show that NO_3_^−^ promoted the synthesis of SGs in stevia leaves by regulating the expression of SGs-related genes combining by key TFs, especially MYB and WRKY (Figure 8).

## 4. Materials and Methods

### 4.1. Plant Material and Experimental Conditions

Field and pot experiments were simultaneously conducted at the Institute of Botany, Jiangsu Province, and the Chinese Academy of Sciences in 2019, from June to September. In the experiments, the similarly sized seedlings of stevia (*Stevia rebaudiana* Bertoni) cultivar Zhongshan No. 8′ were used. The basic properties of the experimental soil used here have been previously described [17], and the total N, Olsen-phosphorus and NH_4_OAc-potassium contents are 2.41 mg g^−1^, 60.91 mg kg^−1^ and 283.22 mg kg^−1^, respectively.

Both field and pot experiments contained two N fertilization regimes of (1) ammonium N (NH_4_^+^) fertilization (in the form of ammonium sulfate) and (2) nitrate N (NO_3_^−^) fertilization (in the form of calcium nitrate). These two treatments only differed in the N fertilizer forms with N fertilization rate and topdressing strategies being identical. The fertilization rates of N, phosphorus and potassium for the field experiment were 300 kg ha^−1^, 75 kg ha^−1^ and 90 kg ha^−1^, respectively. N fertilizers were applied three times, at a ratio of 50%, 30%, 20% separately before transplanting, at the fast-growing stage and late-branching stage, as described previously [16]. Phosphate fertilizers were all applied before transplanting in the form of calcium superphosphate, while potassium fertilizers were applied twice in equal amounts, respectively, before transplanting and at the fast-growing stage. For the pot experiment, all fertilization rates and strategies were consistent with the field experiment, with the specific fertilizer amounts calculated according to the relationship between the pot soil weight and the field soil weight of the cultivated layer (15 cm). In the field experiment, the plots with different fertilization regimes were arranged at intervals to reduce the discrete influence of environmental factors, but this was not done in the pot experiment due to the stable greenhouse conditions.

### 4.2. Sampling and Processing

Plants in field and pot experiments were harvested at the flower bud stage that is defined as the harvest stage in stevia cultivation. Six biological replicates were taken for each treatment. Half of these plants in one treatment (three replicates) were harvested as dry samples, while the rest of the plants were collected as fresh samples. Specifically, three of the replications were cut alongside the stem base and then divided into stems and leaves in the lab after washing with distilled water. Then, all these samples were separately dried at 105 °C for 30 min and then at 70 °C to constant weight, prior to being ground and mixed. Simultaneously, the fresh leaves of the remaining replicates were ground in liquid nitrogen, and then stored in a −80 °C freezer for further analysis. The dried samples were used for the measurements of total N (TN), total carbon (TC), and SG content. The fresh samples were used for the measurements of leaf ammonium and nitrate content, as well as for RNA-seq work.

### 4.3. Measurement of TN, NO_3_^−^ Content, NH_4_^+^ Content in Stevia Leaves

The leaf total N content was measured following the H_2_SO_4_–H_2_O_2_ digestion method of Kjeldahl [73]. The extraction and measurement of NO_3_^−^ followed the method of Cataldo et al. [74], while the measurement of NH_4_^+^ used the method of Lin and Kao [75].

### 4.4. Extraction and Analysis of Leaf Steviol Glycosides (SGs) Content

The detection of leaf SGs content was conducted according to the method of Sun et al. [15], with a slight modification. Briefly, the leaf samples were ground with 80% ethanol and then placed in a boiling water bath for 1 h before centrifugation at 12,000× *g* for 10 min. The supernatants would be then rotary-evaporated and redissolved in distilled water for further high-performance liquid chromatography (HPLC) detection. The HPLC working conditions were kept the same as previously reported, and the contents of different SG components were calculated according to standard curves of Reb A, STV, and Reb C (99.99% pure, ChromaDex, Irvine, CA, USA).

### 4.5. Transcriptome Analysis of Stevia Leaves

The RNA isolation and transcriptome analysis procedures were performed by staff at Beijing BioMarker Technology (Beijing, China), as previously reported [17]. Briefly, total RNA was isolated using RNAiso Plus (Takara Bio Inc., Shiga, Japan). The RNA degradation, purity and integrity were then assessed according to the manufacturer’s instructions. Library preparation for RNA-Seq was generated using the NEBNext^®^ Ultra™ RNA Library Prep Kit for Illumina^®^ (NEB, San Diego, CA, USA), and the library preparations were sequenced using an Illumina HiSeq X-ten platform.

The high-quality clean-read data were obtained by removing reads containing adapter contamination and eliminating the low-quality reads. HISAT 2 program and StringTie were used for the reads mapping and assembly [76,77], with the genome data of stevia referenced for further annotation [29]. Functional annotation of all identified genes was performed through NCBI nonredundant protein sequences, nonredundant nucleotide sequences, SwissProt, Gene Ontology (GO), Clusters of Orthologous Groups of proteins (KOG/COG) and the Kyoto Encyclopedia of Genes and Genomes (KEGG).

### 4.6. Differentially Expressed Genes (DEGs) and Enrichment Analysis

Gene expression levels were represented by the FPKM (fragments per kilobase of exon per million fragments mapped reads) value using RNA-seq data. The DESeq2 was used to calculate the differences in the expression between NH_4_^+^ and NO_3_^−^ treatments. We used a false discovery rate (FDR) of 0.01 and a fold-change of 2 as the threshold for DEGs identification. The subsequently GO and KEGG enrichment analyses were performed based on all of these DEGs, implemented by the GOseq R package-based Wallenius noncentral hypergeometric distribution and KOBAS (2.0) software (center for bioinformatics of Peking University, Beijing, China) [78].

### 4.7. MapMan Analysis

For metabolic pathway analysis, stevia transcripts were annotated and classified into MapMan BINs using plaBi dataBase (https://www.plabipd.de/portal/mercator-sequence-annotation (accessed on 5 March 2021)), and the functional category analysis of DEGs was performed by MapMan version 3.6.0 (http://mapman.gabipd.org/web/guest (accessed on 5 March 2021), Max Planck Institute for Molecular Plant Physiology, Golm, Potsdam, Germany).

### 4.8. Quantitative Real-Time PCR (qRT-PCR) Validation of DEGs

In this study, nine genes involved in SGs synthesis were selected for the verification of the DEG results. As shown in Supplemental Table S4, *Actin* was used as endogenous control and the primers were designed using Primer 3.0 program. qRT-PCR reactions were conducted on an ABI 7500 real-time PCR system using SYBR Green master mix (TaKaRa, Dalian, China) and the relative expression of target genes was calculated by the 2^−ΔΔCt^ method [79].

### 4.9. Data Availability

Data sets of this bio-project (PRJNA745392) are available at the NCBI Sequence Read Archive (SRA) with the accession of SUB9990898. SAMN20165632, SAMN20165633 and SAMN20165634 are the bio-sample names of the control group (A-N), while SAMN20165635, SAMN20165636, and SAMN20165637are those for the treatment group (N-N).

### 4.10. Statistical Analysis

One-way analysis of variance (ANOVA) and two-way ANOVA were respectively used to assess differences for each parameter among treatments and the interaction between treatments and experimental cultures, using the SPSS 16.0 (IBM, Armonk, NYC, USA) statistical software package. Means and calculated standard deviations were reported. Significance was tested at the 5% level.

## 5. Conclusions

Our results showed that NO_3_^−^, rather than NH_4_^+^, can significantly promote SGs synthesis in stevia leaves, without losing leaf biomass. Through transcriptomic analysis, we found that N forms can induce metabolic reprogramming including NO_3_^−^-enhanced terpenoid synthesis. Such influence may be dependent on the activation of the MYB/WRKY TFs on the expressions of key enzymes of terpene synthesis. These represent potential targets to increase SGs via plant breeding via even transgenic or gene-editing approaches. More immediately, the proper use of NO_3_^−^ fertilization seems likely to be an immediate and cost-effective manner to boost SG yield from stevia.

## Figures and Tables

**Table 1:** Effect of different nitrogen forms on the biomass (g plant^−1^), total nitrogen (TN, mg g^−1^ DW) content, total carbon (TC, mg g^−^^1^ DW) content, carbon–nitrogen ratio (C/N), ammonium nitrogen (A-N, mg g^−1^ FW) content and nitrate nitrogen (N-N, mg g^−1^ FW) content in stevia leaves.

Experimental Condition	Treatment	Leaf Biomass	TN	TC	C/N	A-N	N-N
Pot	A-N	2.90 ± 0.18 b	32.43 ± 3.61 b	461.37 ± 13.84 bc	14.36 ± 1.79 b	0.13 ± 0.00 a	0.32 ± 0.02 c
	N-N	2.65 ± 0.12 b	30.14 ± 0.88 b	450.38 ± 15.08 c	14.96 ± 0.94 b	0.12 ± 0.02 a	0.44 ± 0.05 ab
Field	A-N	5.74 ± 0.97 a	25.11 ± 0.75 a	491.04 ± 13.38 a	19.57 ± 0.71 a	0.12 ± 0.00 a	0.34 ± 0.03 bc
	N-N	4.99 ± 1.18 a	24.44 ± 0.44 a	482.86 ± 4.69 ab	19.76 ± 0.30 a	0.12 ± 0.01 a	0.49 ± 0.09 a
N forms		ns	ns	ns	ns	ns	**
Experimental cultures		**	**	**	**	**	ns
N forms * Experimental cultures		ns	ns	ns	ns	ns	ns

Stevia plants growth under pot or field condition were supplied with ammonium-nitrogen (A-N) or nitrate-nitrogen (N-N) at the same N levels. Values represent the means ± SD of three biological replicates. ANOVA results are indicated; different letters indicate significant differences in the same indicator, * and ** indicate significant difference at 0.05 and 0.01 probability levels, respectively; ns means nonsignificant difference. DW: dry weight, FW: fresh weight.

## Data Availability

The data that support the findings of this study are openly available in the NCBI Sequence Read Archive (SRA) under projects PRJNA745392.

## Supplementary Materials

The following are available online at https://www.mdpi.com/article/10.3390/ijms22168549/s1.
