# Supplementary material for: Transcriptomic Characterization of Nitrate-Enhanced Stevioside Glycoside Synthesis in Stevia (Stevia rebaudiana) Bertoni
Source: Int J Mol Sci. 2021 Aug 9;22(16):8549. doi: 10.3390/ijms22168549 (PMC8395231; doi:10.3390/ijms22168549)
Supplement: Supplementary file 1 [file ijms-22-08549-s001.zip › ijms-1317603-supplementary.pdf]

Supplemental Material

Supplemental Table S1 Summary of the transcriptomic data

Item	A-N	N-N
Raw reads(bp)	42,888,959	51,454,116
Clean reads(bp)	21,444,479	25,727,058
Clean bases(G)	6.41	7.70
Clean GC(%)	46.19	45.42
Clean Q30(%)	95.37	95.27
Total mapped	60.45	76.30
Uniquely mapped	48.83	62.55
Multiply mapped	11.62	13.75
Unmapped reads	26.47	33.96
Number of detected genes in libraries	35,424	36,063

A-N: ammonium nitrogen; N-N: nitrate nitrogen

Supplemental Table S2 Summary of the relationship between WRKY transcription factors and plant terpenoid metabolism as indicated in previous publications

Plant species	Tissue	Terpenoid	WRKY TF(s)	Effect	References
Artemisia annua	Trichome	Artemisinin	AaWRKY1; AaWRKY9	Positive	Han et al. 2014; Fu et al. 2021
Conyza Blinii	Fruit	Saponin	CbWRKY24	Positive	Sun et al. 2018
Panax ginseng	Root	Ginsenoside	PgWRKY4x	Positive	Yao et al. 2020
Salvia miltiorrhiza	Hairy roots	Tanshinone	SmWRKY2 SmWRKY1	Positive	Deng et al. 2019; Gao et al. 2018
Salvia Sclarea	Root	Abietane diterpenes	AtWRKY18; AtWRKY40	Positive	Alfieri et al. 2018
Withania somnifera	Leaf	Triterpenoids	WsWRKY1	Positive	Singh et al. 2017

References

Alfieri M, Vaccaro M C, Cappetta E, et al. Coactivation of MEP-biosynthetic genes and accumulation of abietane diterpenes in *Salvia sclarea* by heterologous expression of WRKY and MYC2 transcription factors. ***Scientific Reports***, 2018, 8, 1-13.

Cao W, Wang Y, Shi M, et al. Transcription factor *SmWRKY1* positively promotes the biosynthesis of tanshinones in *Salvia miltiorrhiza*. ***Frontiers in Plant Science***, 2018, 9, 554.

Deng C, Hao X, Shi M, et al. Tanshinone production could be increased by the expression of SmWRKY2 in Salvia miltiorrhiza hairy roots[J]. ***Plant Science***, 2019, 284: 1-8.

Fu X, Peng B, Hassani D, et al. *AaWRKY9* contributes to light‐ and jasmonate‐ mediated to regulate the biosynthesis of artemisinin in *Artemisia annua*. ***New Phytologist***, 2021.

Han J, Wang H, Lundgren A, et al. Effects of overexpression of *AaWRKY1* on artemisinin biosynthesis in transgenic *Artemisia annua* plants. ***Phytochemistry***, 2014, 102: 89-96.

Singh AK, Kumar SR, Dwivedi V, et al. A WRKY transcription factor from *Withania somnifera* regulates triterpenoid withanolide accumulation and biotic stress tolerance through modulation of phytosterol and defense pathways. ***New Phytologist***, 2017, 215, 1115-1131.

Sun W J, Zhan J Y, Zheng T R, et al. The jasmonate-responsive transcription factor *CbWRKY24* regulates terpenoid biosynthetic genes to promote saponin biosynthesis in *Conyza blinii* H. Lév. ***Journal of Genetics***, 2018, 97, 1379-1388.

Yao L, Wang J, Sun J, et al. A WRKY transcription factor, *PgWRKY4X*, positively regulates ginsenoside biosynthesis by activating squalene epoxidase transcription in *Panax ginseng*. ***Industrial Crops and Products***, 2020, 154: 112671.

Supplemental Table S3 Summary of the relationship between MYB transcription factors and plant terpenoid metabolism as indicated in previous publications

Plant species	Tissue	Terpenoid	MYB TF(s)	Effect	References
Artemisia annua	Trichome	Artemisinin	AaMYB17; AtMYB61	Positive	Qin et al. (2021); Matías-Hernández et al. (2017)
Artemisia annua	Trichome	Artemisinin	AaMYB15	Negative	Wu et al. 2021
Citrus	Leaf	Limonoids	CiMYB42	Positive	Zhang et al. 2020
Salvia miltiorrhiza	Hairy/roots	Tanshinone	SmMYB98; SmMYB9b; SmMYB36	Positive	Hao et al. 2020; Zhang et al. 2017; Ding et al. 2017
Spearmint	Leaf	Monoterpene	MsMYB	Negative	Reddy V A. 2017
Tomato	Leaf/fruit	Beta-amyrin; Terpene volatiles	VvMYB5b; SlMYB75	Positive	Mahjoub et al. 2009; Jian et al, 2019
Tomato	Trichome	Sesquiterpene	SlMYB75	Negative	Gong et al. 2021

References

Ding K, Pei T, Bai Z, et al. *SmMYB36,* a novel R2R3-MYB transcription factor, enhances tanshinone accumulation and decreases phenolic acid content in *Salvia miltiorrhiza* hairy roots. ***Scientific Reports***, 2017, 7, 1-15.

Gong Z, Luo Y, Zhang W, et al. A SlMYB75-centred transcriptional cascade regulates trichome formation and sesquiterpene accumulation in tomato. ***Journal of Experimental Botany***, 2021, 72, 3806-3820.

Hao X, Pu Z, Cao G, et al. Tanshinone and salvianolic acid biosynthesis are regulated by *SmMYB98* in *Salvia miltiorrhiza* hairy roots. ***Journal of Advanced Research***, 2020, 23, 1-12.

Jian W, Cao H, Yuan S, et al. *SlMYB75*, an MYB-type transcription factor, promotes anthocyanin accumulation and enhances volatile aroma production in tomato fruits. ***Horticulture Research***, 2019, *6*, 1-15.

Mahjoub A, Hernould M, Joubès J, et al. Overexpression of a grapevine R2R3-MYB factor in tomato affects vegetative development, flower morphology and flavonoid and terpenoid metabolism. ***Plant Physiology and Biochemistry***, 2009, 47, 551-561.

Matías-Hernández L, Jiang W, Yang K, et al. Aa MYB 1 and its orthologue *AtMYB61* affect terpene metabolism and trichome development in *Artemisia annua* and *Arabidopsis thaliana*. ***The Plant Journal***, 2017, 90, 520-534.

Qin W, Xie L, Li Y, et al. An R2R3-MYB transcription factor positively regulates the glandular secretory trichome initiation in *Artemisia annua* L. ***Frontiers in Plant Science***, 2021, *12*, 560.

Reddy V A, Wang Q, Dhar N, et al. Spearmint R2R3-MYB transcription factor *MsMYB* negatively regulates monoterpene production and suppresses the expression of geranyl diphosphate synthase large subunit (MsGPPS. LSU). ***Plant Biotechnology Journal***, 2017, 15, 1105-1119.

Wu Z, Li L, Liu H, et al. *AaMYB15*, an R2R3-MYB TF in *Artemisia annua*, acts as a negative regulator of artemisinin biosynthesis. ***Plant Science***, 2021, 308, 110920.

Zhang J, Zhou L, Zheng X, et al. Overexpression of *SmMYB9b* enhances tanshinone concentration in *Salvia miltiorrhiza* hairy roots. ***Plant Cell Reports***, 2017, 36, 1297-1309.

Zhang P, Liu X, Yu X, et al. The MYB transcription factor *CiMYB42* regulates limonoids biosynthesis in citrus. ***BMC Plant Biology***, 2020, 20, 1-12.

Supplemental Table S4 The primers used for qRT-PCRs in this study

Gene	Primers (forward primer, F and reverse primer, R) (5`-3`)	Accession no.
SrDXR	F:TCCGAACTCGTAACTGCACC<br>R:CAAGAGCTACCGCGGTCAAA	AJ429233.1
SrMDS	F:ACCGAAACACACAGTGAGCA<br>R:TGAGAGGGTATCCAGGCTCC	DQ631427
SrIDI	F:TTCATTGTGCGAGACGTTGC<br>R:TGAGTTGAGGGGCATATGTGG	DQ989585
SrGGDPS	F:AAACACCAATCAAACCCATCAGAA<br>R:GAACCGAGGCGTCCAGAG	DQ432013
SrKS1-1	F: GAAGAAGAGGTTGTGGAGGA<br>R: CCCGTCATCGTTTGCGTAA	AF097310.1
ent-KAH	F: TGTTATCGTATCTCGGTCCTGAT<br>R: CTTCCTTCCCTCGCCAATG	DQ398871
SrUGT85C2	F:CGAGCATCCCAATCAGAGAATC<br>R:CAGGCAGCAAGTGTCCAAT	AY345978.1
SrUGT74G1	F: TCTACTCATCCCATTCCCTTTACA<br>R: CCGCCTTCATCACAACCAT	AY345982
SrUGT76G1	F: CGAGCATCCCAATCAGAGAATC<br>R: CAGGCAGCAAGTGTCCAAT	AY345978
ACTIN	F: CACCGAAGCACCTCTCAATC<br>R: CCATCTCCAGAATCCAACACAAT	AF548026

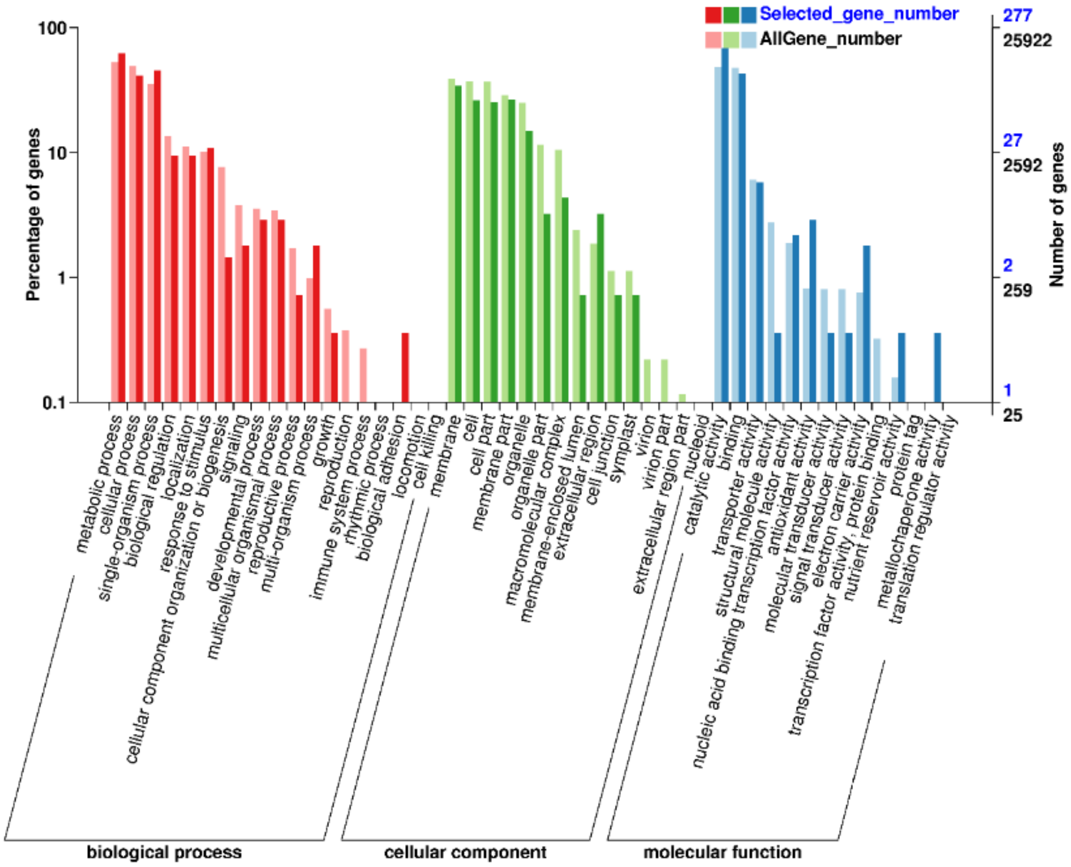

Supplemental Fig. S1 Gene ontology annotation of the differentially expressed genes (DEGs) between the ammonium and nitrate fed stevia plant. The x-axis represents different subcategories while the y-axis indicates the number of genes with the corresponding GO terms.

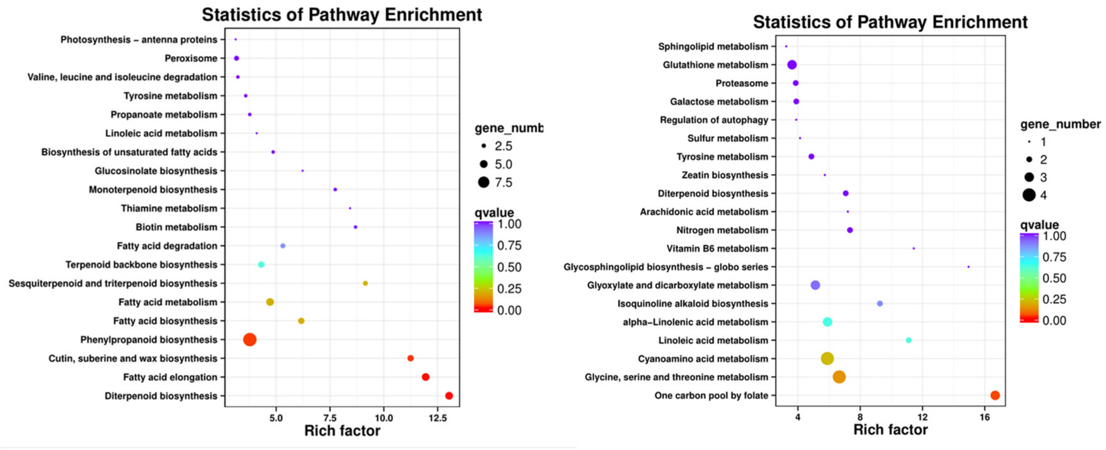

Supplemental Fig. S2 Kyoto encyclopedia of genes and genomes (KEGG) enrichment analysis of the up-regulated (A) and down-regulated (B) DEGs between the ammonium and nitrate fed stevia plants.

The x-axis represents the enrichment factor while the y-axis indicates the name of KEGG pathway. The color of each circle refers to the q-value while the size indicates the gene numbers mapped to the pathway.

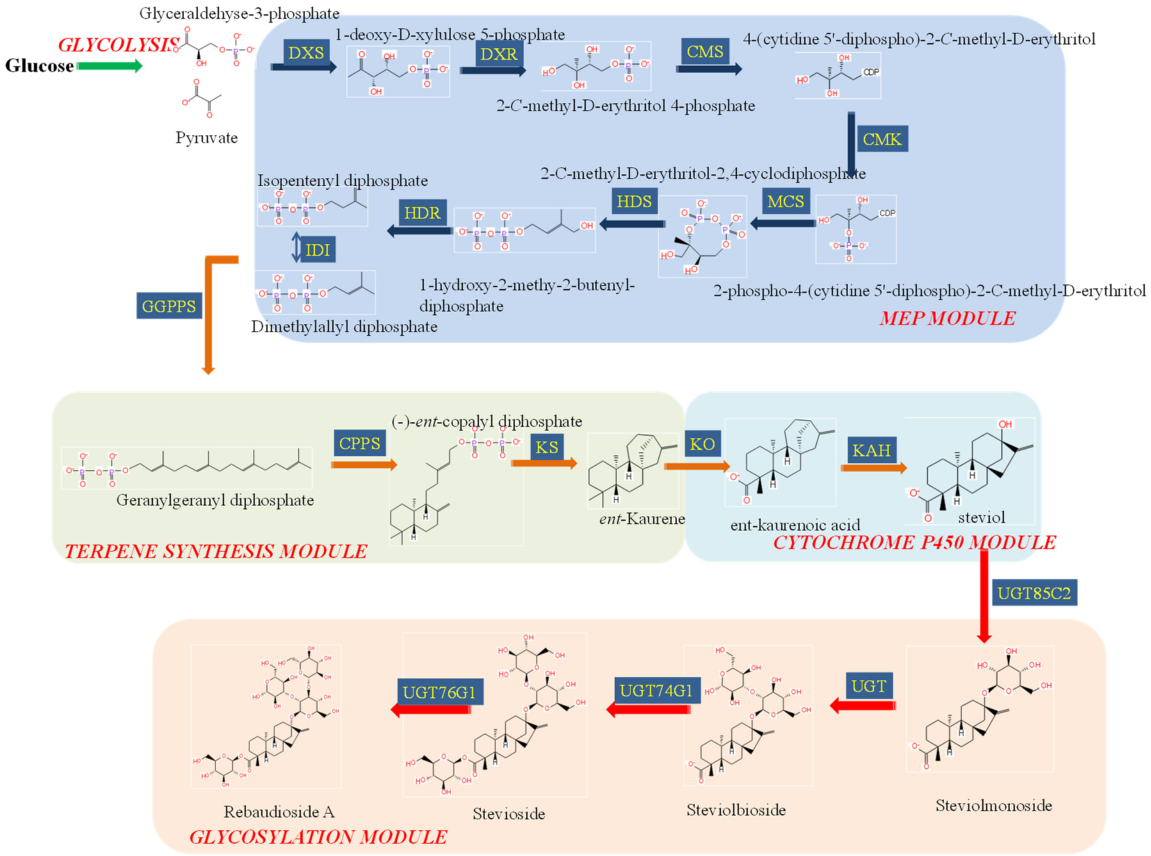

Supplemental Fig. S3 Diagrammatic representation of the steviol glycoside (SG) biosynthesis pathway

Abbreviations: DXS (1-deoxy-D-xylulose-5-phosphate synthase); DXR (1-deoxy-D-xylulose 5-phosphate reductoisomerase), CMS (2-C-methyl-D-erythritol 4-phosphate cytidylyltransferase), CMK (4-diphosphocytidyl-2-C-methyl-D-erythritol kinase), MCS (2-C-methyl-D-erythritol 2,4-cyclodiphosphate synthase), HDS (4-hydroxy-3-methylbut-2-enyl diphosphate synthase), HDR (4-hydroxy-3-methylbut-2-enyl diphosphate reductase), IDI (isopentenyl-diphosphate delta-isomerase), GGPPS (geranylgeranyl pyrophosphate synthase), CPPS (ent-copalylpyrophosphate synthase), KS (ent-copalyl diphosphate synthase), KO (ent-kaurene oxidase), KAH (ent-kaurenoic acid hydroxylase), UGT85C2 (UDP-glycosyltransferase 85C2), UGT74G1 (UDP-glycosyltransferase 74G1), UGT76G1 (UDP-glycosyltransferase 76G1).
